# Revisiting Hebb: The Mechanisms of Repetition Learning

**DOI:** 10.1177/17456916251408052

**Published:** 2026-01-30

**Authors:** Philipp Musfeld, Klaus Oberauer

**Affiliations:** 1 Psychological Methods, Department of Psychology, University of Amsterdam; 2 Cognitive Psychology, Department of Psychology, University of Zurich

**Keywords:** working memory, long-term memory, repetition learning, Hebb effect

## Abstract

In 1961, Donald Hebb established a classic paradigm for studying repetition learning: He asked participants to remember several memory sets for an immediate serial recall task and repeated one set multiple times throughout the experiment. Participants’ ability to recall the repeated set improved gradually with repetitions, thereby demonstrating repetition learning. Explaining this effect has concerned researchers for decades because it provides key insights into how we form durable memory representations through repeated exposure. In this article, we revisit the dominant views on the mechanisms underlying repetition learning, thereby challenging the central assumption that repetition learning is gradual and implicit. We show how these views have emerged from flawed analytical approaches, summarize recent evidence strongly contradicting these claims, and reanalyze previously published data to illustrate how correcting implausible analytical assumptions leads to different theoretical conclusions. We propose an updated theoretical framework of the cognitive mechanisms underlying repetition learning that integrates elements from previous models of the Hebb repetition effect with established models of episodic memory, thereby joining two branches of the memory literature.

When we attempt to acquire new knowledge, we frequently engage in repeated practice. We study new words over and over when we learn the vocabulary of a new language, and we repeatedly rehearse what we want to say when preparing for a presentation. Learning from repetition is fundamental to human knowledge acquisition, and the cognitive mechanisms underlying this process have concerned researchers for more than 60 years.

In 1961, Donald Hebb established an experimental paradigm for the study of repetition learning. He presented participants with sequences of nine digits and asked them to recall a sequence immediately after the last digit had been presented. Unbeknownst to participants, he repeated one sequence multiple times throughout the experiment. Hebb observed that participants’ ability to recall the repeated sequence improved steadily with the number of repetitions, whereas performance on interleaving unique sequences remained constant (Hebb, 1961; for a schematic representation of the paradigm and findings, see Figs. 1a and 1b). Hebb himself was surprised by this result. He had not expected to observe any learning effects when interrupting the repetition of a sequence by other unique filler sequences. He had hypothesized that the immediate serial recall of a memory sequence was solely based on an “activity trace,” a reverberatory activation of the encoded information (Hebb, 1949). Once a new sequence is presented, this activity trace would get wiped out, leading to complete forgetting of the previous sequence. His results proved him wrong and had important implications for our understanding of memory and learning. Hebb showed that even a single presentation of a sequence must lead to a long-lasting structural change in the brain, allowing us to cumulatively learn from repeated experiences (Hebb, 1961).

**Fig. 1.**
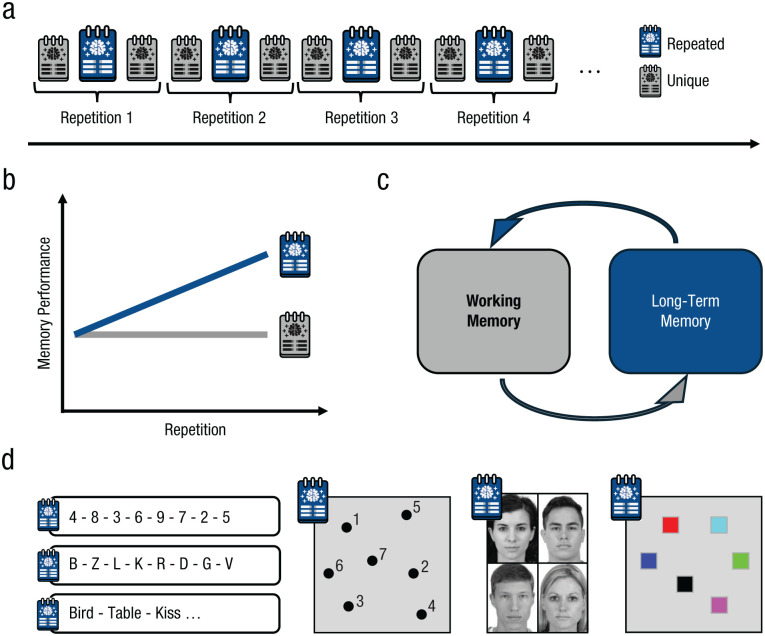
Trial structure of a typical Hebb paradigm (a) in which one memory set is repeated amid unique filler sets. Schematic visualization of the data pattern typically observed in the Hebb paradigm (b). Memory performance for the repeated memory set steadily increases with repetitions, whereas memory performance for unique, unrepeated filler sets remains constant. Schematic visualization of the bidirectional flow of information between working memory and long-term memory (c) used to explain the increase in memory performance observed in the Hebb repetition effect. Examples of different stimulus materials for which the Hebb repetition effect has been demonstrated (d). Face stimuli were taken from the Chicago Face Database (Ma et al., 2015).

Today, more than 60 years after Hebb’s seminal article, the *Hebb repetition effect* has been replicated numerous times and continues to inform our understanding of human memory and learning for various reasons. First, the Hebb repetition effect offers a model system for understanding the bidirectional interaction between working memory and long-term memory. It demonstrates (a) how repeatedly representing the same information in working memory can lead to the formation of long-lasting representations in long-term memory and (b) how these representation in long-term memory can, in turn, support the maintenance of information in working memory (Fig. 1c; Burgess & Hitch, 2006; Page & Norris, 2009). Second, the Hebb repetition effect has been proposed as an analogue of word-form learning and thus can provide important insights into the mechanisms underlying language acquisition (Gupta, 2008; Norris et al., 2018; Page & Norris, 2008; Saint-Aubin & Guérard, 2018; Szmalec et al., 2009, 2012). Third, the Hebb repetition effect has been replicated across many different stimulus domains and presentation modalities, including sequences of letters (e.g., Araya et al., 2022; Page et al., 2006), words (e.g., Dutli et al., 2024; Page et al., 2013), spatial locations (e.g., Couture & Tremblay, 2006; Gagnon et al., 2005; Tremblay & Saint-Aubin, 2009; Turcotte et al., 2005), and faces (Horton et al., 2008; A. J. Johnson & Miles, 2019; A. J. Johnson et al., 2017), as well as simultaneously presented visual arrays (Musfeld, Souza, & Oberauer, 2024; Musfeld et al., 2023; Shimi & Logie, 2019; Souza & Oberauer, 2022), highlighting its universality as a very general form of learning. Thus, understanding the cognitive mechanisms underlying the Hebb repetition effect is informative about many basic functionalities of our cognitive system.

In this article, we revisit the prevailing assumptions that have been proposed to explain learning in Hebb’s paradigm over the last decades and reevaluate their validity in light of recent findings and developments. We demonstrate that some of these assumptions are flawed and show how they have resulted from incorrect ways of analyzing data. On the basis of these new insights, we propose a new theoretical framework of the mechanisms underlying repetition learning in the Hebb paradigm, thereby offering a new perspective on one of the most classical effects in cognitive psychology.

## Prevailing Assumptions About the Mechanisms Underlying Repetition Learning

The question of how we learn from repeated exposure involves two theoretically relevant aspects. The first concerns the flow of information from working memory to long-term memory, that is, how repeated exposure to the same information in a working memory task leads to a long-lasting representation in long-term memory. The second concerns the flow of information from long-term memory to working memory, that is, how the representation in long-term memory can be beneficial to performance in a test of working memory. Several attempts have been made to explicate these mechanisms in computational models of memory (Burgess & Hitch, 2005, 2006; Page & Norris, 2009). Although these models show substantial differences in their general conception of short-term and working memory, they nevertheless share some common assumptions about the mechanisms underlying repetition learning.

The first of these common assumptions is the idea that repetition learning leads to the formation of new chunk representations in long-term memory. Chunk formation is a process by which multiple separate elements of information are integrated into one unified representation (i.e., a “chunk”) of the elements and their structure (Cowan & Chen, 2008; Ericsson & Kintsch, 1995; Miller, 1956). Related to the Hebb repetition effect, this can be the integration of a sequence of digits into a phone number (Hebb, 1961; McKelvie, 1987), the integration of phonemes into a word (Szmalec et al., 2009, 2012), or the integration of multiple pieces on a chess board into a known pattern (Ericsson & Kintsch, 1995; Gobet & Simon, 1996a, 1996b). Chunk formation is a key feature for dealing with the limited capacity of our cognitive system because it allows us to represent information in a more efficient or compressed way, thereby making room for other information to be processed (Awh & Vogel, 2025; Brady et al., 2009; Chekaf et al., 2016; Huang & Awh, 2018; Ngiam et al., 2019; Norris & Kalm, 2021; Norris et al., 2020; Thalmann et al., 2019).

A second assumption that can be identified is that repetition learning reflects a cumulative process by which every repetition gradually strengthens a representation in memory (Burgess & Hitch, 2005; Page & Norris, 2009). This learning process is initiated by the first presentation of a repeated memory set and is commonly assumed to be rather slow, with each repetition leading to a gain in recall accuracy of only some single-digit percentage.

The last assumption that has been proposed consistently over the last decades is the claim that the Hebb repetition effect is an example of implicit learning. This means that participants do not have to be aware of studying the same information over and over again for learning^1^ to occur. Although this assumption is not inherently included in the computational models of the Hebb repetition effect, it is a widely accepted assumption that has been supported by several empirical findings (Couture & Tremblay, 2006; Guérard et al., 2011; Hebb, 1961; McKelvie, 1987; Seger, 1994; Smalle et al., 2018). In the following sections, we revisit the evidence for these three assumptions and evaluate their validity in light of new findings and recent developments.

### Assumption 1: repetition learning forms chunks

The assumption that Hebb repetition learning leads to the formation of chunked representations has evolved primarily from studies demonstrating conditions under which repetitions did not result in learning, thereby revealing key constraints on what is learned. For instance, Schwartz and Bryden (1971) reported no Hebb repetition effect when the first two items of an otherwise repeated sequence varied on each presentation, and Hitch et al. (2005) found that repeating only every other item (e.g., only odd or even positions) also did not result in any learning. Similarly, Cumming et al. (2003) observed no transfer of learning when only every other item of a once well-learned list was repeated. These results converge on the idea that learning in the Hebb paradigm is *not* operating at the level of single elements; rather, it seems to rely on integrating the elements of a memory set into a unified representation—a chunk that is stored and retrieved as a single unit (Cumming et al., 2003; Hitch et al., 2005; Page & Norris, 2009).

Recent evidence points to the possibility that chunk formation might be more flexible than initially assumed. Mızrak and Oberauer (2022), for example, demonstrated that participants could selectively retrieve subsets of a previously learned list in isolation, suggesting that retrieval of the acquired long-term memory representation does not necessarily unfold in an all-or-nothing manner. This aligns with recall-time patterns in visual repetition learning (Adam et al., 2024): After several repetitions of the same six-item array, the times of successive recall responses peak after the first and fourth response, suggesting that elements of a learned memory set are retrieved from long-term memory in two subsets of three items.

There is evidence that not only the retrieval but also the learning itself operates on smaller subsets of a memory set. For instance, early work by Bower and Winzenz (1969) and Winzenz (1972) showed that the Hebb effect is susceptible to grouping effects: When memory lists were grouped by temporal gaps, repetition learning only appeared when the grouping structure remained consistent across trials. When the grouping structure was changed with every repetition, learning was absent, even though all list items were repeated. More recently, Musfeld, Dutli, et al. (2024) demonstrated that a Hebb repetition effect could also be observed when only a contiguous subset of list items was repeated, provided that participants were able to identify the beginning and end of that subset. These results suggest that learning does not have to result in chunks that integrate the entire memory set. The learning mechanism appears to be flexible enough to acquire smaller chunks integrating subsets of the memory set (see also Szmalec et al., 2009, 2012). These subsets are likely to be established already during initial encoding and then continue to guide chunk learning over consecutive repetitions (Musfeld, Dutli, et al., 2024). This is consistent with interresponse time patterns observed in free recall, showing that participants spontaneously break down longer lists into smaller, more manageable subsets (N. R. Greene et al., 2025).

A challenge for the chunking hypothesis comes from the Hebb repetition effect with complex span tasks (Araya et al., 2022, 2024; Oberauer et al., 2015). In a complex span task, the presentation of each memory item is interleaved with the processing of one or more distractor stimuli, thereby potentially disrupting the integration of repeated items into a chunk (Hitch et al., 2005; Musfeld, Dutli, et al., 2024). This seems to contradict the idea that Hebb repetition learning relies on integrating contiguous sequences of items into chunks. However, distractor stimuli do not have to be memorized and can be removed from working memory immediately after processing (Oberauer & Lewandowsky, 2016). In that way, they do not become part of the memory representation of the list. After distractors are removed from working memory, the list items are again represented as a list of contiguous items that can be chunked. This is consistent with Araya et al. (2022, 2024), who found that repetition learning in simple and complex span tasks is based on the same mechanism, which is likely to be chunking.

One benefit of chunking is that it allows us to represent information in a more efficient or compressed way, thereby freeing up capacity in working memory for other information to be maintained and processed (Bartsch & Shepherdson, 2022; Mızrak & Oberauer, 2022; Norris & Kalm, 2021; Thalmann et al., 2019). For example, when asked to remember the sequence “PDF-KGW-HPN,” participants remember not only the preknown chunk “PDF” but also the unfamiliar letter sequence following it better than when no chunk is present early in the sequence. We demonstrated that representations learned through repetition can free up capacity in working memory as well (Musfeld, Dutli, et al., 2024), consistent with this characteristic of chunks. By using partially repeated memory lists in which only a specific subset of a memory list contained repeating items, we observed that learning of the repeated subset improved memory not only for the repeated items but also for the other items within the same list that were not repeated. This finding shows that learning the repeated subset reduces the load on working memory, thereby freeing up capacity for remembering other information.

Taken together, the assumption that repetition learning leads to the formation of chunks is empirically well supported. Yet what exactly constitutes a chunk remains surprisingly elusive (Gobet et al., 2016; Terrace, 2001). In the literature, the term “chunking” has been used with a wide range of meanings (Allen et al., 2021; Anderson, 1993; French et al., 2011; Gobet & Simon, 1996b; Gobet et al., 2016; Huang & Awh, 2018; N. F. Johnson, 1970, 1972; Jones et al., 2014; Norris & Kalm, 2021; Orbán et al., 2008; Thalmann et al., 2019), highlighting the need for a clearer and more consistent understanding of chunking to properly understand what kind of representation exactly is learned from repetition.

### Assumption 2: repetition learning reflects a gradual strengthening of representations in long-term memory

The assumption of repetition learning reflecting a gradual learning process is a conclusion inferred from the data pattern observed in almost every experiment conducted on the Hebb repetition effect: When data are aggregated over participants, the results show a gradual improvement in immediate memory performance on the repeated memory set as a function of repetitions (see Fig. 1b). However, this does not guarantee that learning proceeds gradually because the aggregated learning curve is not always a correct reflection of the individual learning curve (Estes, 1956; Gallistel et al., 2004; Hayes, 1953).

**Fig. 2.**
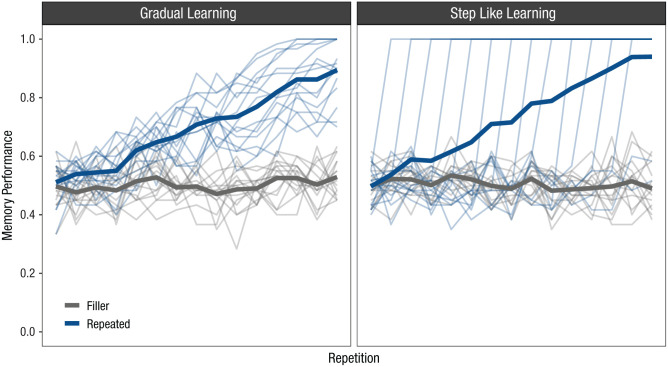
Visualization of two almost identical aggregated learning curves derived from completely different data-generating processes. Data shown in the left panel were simulated by assuming a gradual learning process by which memory performance gradually improves as a function of repetition, and participants differ in their rate of learning. Data shown in the right panel were simulated by assuming a step-like learning process by which a memory set is learned within a single repetition, but participants differ in their onset point of learning. Thin lines represent individual participants; thick lines represent their average.

This problem is illustrated in Figure 2, which presents two extreme scenarios. Figure 2 (left) shows data simulated on the basis of an assumed gradual learning process.^2^ The thin transparent lines show learning curves from individual participants, and the thick line represents their average. This is how one would usually imagine the individual learning curves to look like when drawing conclusions from the aggregated curve: Participants differ in their rate of learning, but for everyone, performance improves gradually over repetitions. Figure 2 (right) shows data simulated on the basis of a different data-generating process in which all participants learn the repeated list within a single repetition but differ in their onset point of learning. Although this reflects a completely different assumption about learning, the resulting aggregated curve looks exactly the same: It suggests a gradual improvement in performance as a function of repetition. Hence, analyzing data aggregated over participants can be misleading, and individual data patterns should be considered for better understanding the processes underlying repetition learning.

We recently addressed this issue by introducing a new Bayesian hierarchical measurement model that allowed us to analyze data on the level of individual participants, rather than the sample average, and to determine the shape of the underlying learning curve more accurately (Musfeld et al., 2023). The model is built on two assumptions. First, it includes two main parameters that determine the shape of the learning curve: one parameter for the rate of learning and an additional parameter for the onset of learning, both of which are freely estimated for every participant. This gives the model full flexibility in capturing the shape of the individual learning curves from a slow but steady increase in performance starting from the first repetition to a rather steep increase in performance with varying onset points of learning—and everything in between. Second, it involves a classification process that determines whether a participant showed a learning effect or not. This process is based on the observation that within a sample of a typical Hebb study many participants’ performance on the repeated list never improves. By separating participants who show a learning effect from those who do not, it can be ensured that the estimation of learning-related parameters is informed only by participants who show a learning effect. The result is a mixture model that (a) includes submodels to differentiate learning from nonlearning participants and (b) estimates parameters reflecting the proportion of learning participants within a sample.

Here, we applied this model to two large data sets to obtain an accurate estimation of the shape of the learning curves.^3^ One data set contained data from approximately 300 participants performing a visual Hebb paradigm in which a visual array of six colored squares was repeated 30 times amid unrepeated filler arrays (for an example, see Fig. 1d). The data were taken from Musfeld et al. (2023). The other data set contained data from approximately 300 participants performing a typical verbal Hebb paradigm in which a sequence of nine consonants was repeated 20 times amid unrepeated filler lists for an immediate serial recall task. These data were collected for the purpose of this article.^4^

**Fig. 3.**
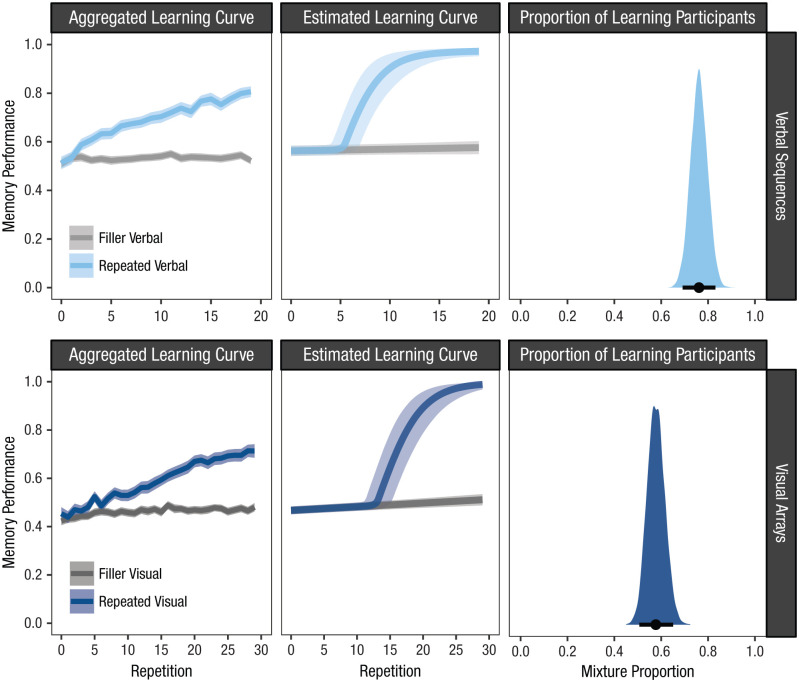
Results from a verbal and visual Hebb experiment. The plots in the left panels show memory performance as a function of repetitions, aggregated over participants. The plots in the middle and right panels show the results of the Bayesian hierarchical mixture model fitted to the same data. The middle panels show the estimated learning curve for the two experiments based on the shape of the underlying individual learning curves (i.e., combining the average onset point of learning with the average rate of learning). The right panels show the estimated mixture proportion, which reflects the proportion of participants who showed a learning effect in the two samples. Shaded areas around the aggregated and estimated learning curves reflect 95% within-subjects confidence intervals and 95% highest density intervals, respectively.

The aggregated results from both experiments are presented in the left panels of Figure 3. Both show the typical data pattern of the Hebb repetition effect: Memory performance gradually increased with repetitions. When looking at the results from the Bayesian hierarchical mixture model, however, a different picture emerges. The middle panels of Figure 3 show the shape of the estimated learning curve based on the underlying individual data patterns for both experiments (i.e., participants’ average onset point of learning combined with participants’ average rate of learning). The right panels of Figure 3 show the estimated proportion of learning participants in the two experiments. What becomes evident from these results is that immediate recall performance on the repeated memory set did not steadily improve with each repetition. Instead, the estimated learning curves show a delay in when the learning process began. In addition, once initiated, the curves are steeper than suggested from the aggregated data. Comparing the learning effects between the two experiments also makes clear that they mainly differ only in (a) the proportion of participants who learned the repeated memory set and (b) the onset point of the learning effect. Once initiated, however, learning seemed to have proceeded at similar rates.

These results contradict the commonly accepted assumption of repetition learning being a continuous process in which each repetition gradually improves memory. This assumption seems to rest on the analysis of aggregated learning curves, which do not resemble the underlying individual curves. When describing learning effects on the level of individuals it becomes evident that repetitions do not always result in learning (i.e., improvement in immediate recall performance). Instead, there seems to be a precondition that must be met for learning to occur. Repetitions seem to improve immediate recall performance only when this condition is met. However, this improvement is much faster than implied by the aggregated learning curve and quickly results in perfect memorization of the entire memory set. If this condition is not met, however, learning can be delayed or does not occur at all. Overall, this implies that not every repetition has the same effect on long-term memory and raises the question under which conditions repetitions strengthen existing memory representations. We propose an answer in the next section.

### Assumption 3: repetition learning occurs implicitly

The assumption of repetition learning being an implicit process dates back to Hebb himself. Although he had not systematically tested whether learning was affected by participants’ awareness of the repetition, his account to explain the observed learning effect included the assumption that repetition awareness was not necessary for learning to occur (Hebb, 1961). Later, several empirical findings supported Hebb’s claim by showing that a Hebb repetition effect could be observed regardless of whether participants reported awareness of a repetition or not (Couture & Tremblay, 2006; Guérard et al., 2011; McKelvie, 1987).

Other researchers have argued that repetition learning requires explicit recognition of what is being repeated (Bower & Winzenz, 1969; Cohen & Johansson, 1967; Cunningham et al., 1984; Winzenz, 1972). Although several studies have supported this idea (e.g., Ngiam et al., 2019; Sechler & Watkins, 1991; Shimi & Logie, 2019; Sukegawa et al., 2019), the dominant view has remained that repetition learning in the Hebb paradigm does not require repetition awareness.

The learning curves observed for individual participants, however, raise questions that challenge this assumption once more: Why do some participants never learn to recall the repeated memory set better? Why can the onset of such learning be delayed but then still result in rapid improvements? One possibility is to assume that repetition awareness is indeed a necessary condition for memory representations to become stronger, and thus, for proper learning to occur. In this case, memory representations are not passively strengthened by every repetition of the repeated list but only once participants explicitly identify a memory set as repeating. As long as the repetition goes unnoticed, no learning occurs, resulting in a delay in the onset, or even the complete absence, of learning.

On the basis of these considerations, we revisit the relationship between learning and awareness in the Hebb paradigm. We start by reevaluating previous empirical evidence claiming that repetition learning is not influenced by repetition awareness and then turn to discussing whether repetition awareness should be considered a necessary condition for repetition learning to occur.

#### Is repetition learning affected by repetition awareness?

Several studies have claimed that the Hebb repetition effect is not affected by whether people are aware of the repetition or not. To our knowledge, there are three studies that came to this conclusion: one that used sequences of digits (McKelvie, 1987) and two that used sequences of spatial locations (Couture & Tremblay, 2006; Guérard et al., 2011). To assess the effect of repetition awareness on repetition learning, these studies classified participants as either aware or unaware and compared the learning effect between the two awareness groups. The classification was based on a postexperimental assessment in which participants were asked whether they noticed anything special about the experiment. Participants were classified as “aware” if they mentioned something about a repetition and as “unaware” if they did not. Here we present a reanalysis of all three studies^5^ by using two different analytical approaches: the one that was used in the original studies and a slightly different approach for which we argue provides a more accurate way of analyzing the data.

**Fig. 4.**
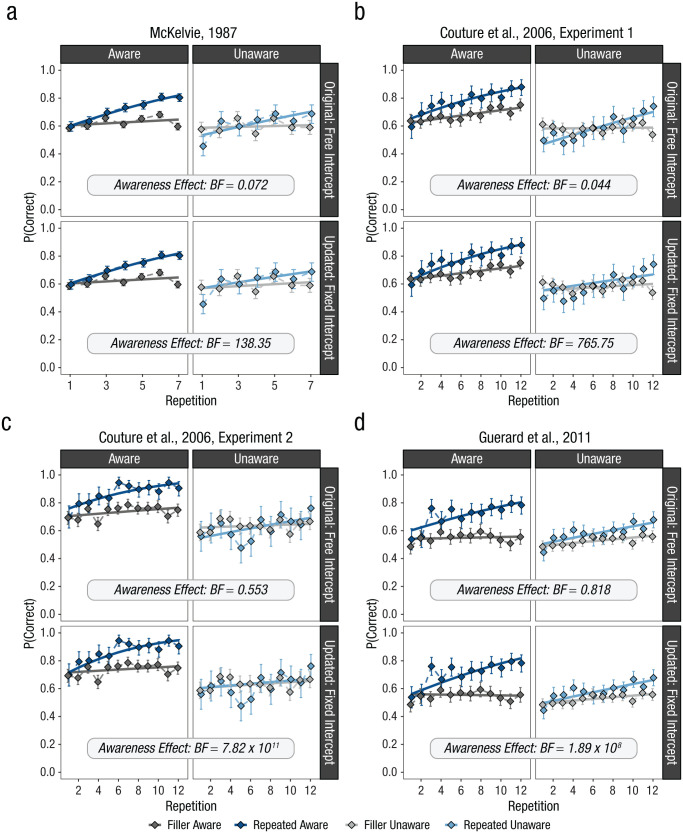
Reanalysis of four experiments (a-d) reporting evidence against an effect of repetition awareness on learning. The top panels of the plots show the results based on the original analysis; the bottom panels show the results based on an alternative reanalysis. Solid lines show the fit of the binomial regression models to the data. Error bars reflect 95% quantile intervals from the binomial distribution. Bayes factors (BFs) indicate the evidence in favor of a three-way interaction between trial type (filler vs. repeated), repetition, and awareness, which reflects the difference between the slopes of the increase in memory performance over repetitions for aware and unaware repeated trials.

Figure 4 shows the results for all three studies, with the data from the aware participants in the left panels of the plots and the data from the unaware participants in the right panels. A substantial difference in the learning effect between the two awareness groups across all studies is clear, yet all studies concluded that learning was not affected by repetition awareness. How does this align? The reason is related to the way the data were analyzed: In all three studies, the learning effect was not estimated in relation to a common baseline (i.e., the performance in nonrepeated filler trials). The Hebb repetition effect, however, is defined as the selective improvement in immediate memory performance on a repeated memory set beyond any changes in immediate memory performance on unique filler sets. Thus, the filler sets serve as a critical baseline that should be considered when estimating the learning effect.

One consequence resulting from this consideration is that memory performance on repeated and unique memory sets should be equalized at the beginning of the experiment because at the very first presentation of the repeated set it is not yet repeated. If this is not considered, the estimated slopes can be biased in two possible ways. In some experiments, the starting point of the regression line for repeated sets in the unaware group was estimated below the baseline performance in corresponding filler sets (see unaware groups in Figs. 4a through 4c), which increased the slope for the unaware repeated sets, although performance never substantially improved above performance in filler sets. In other experiments, however, the starting point of the regression line for repeated sets in the aware group had already started above the baseline performance in corresponding filler sets (see the aware groups in Figs. 4c and 4d), which decreased the slope for the repeated sets, although performance did substantially improve above performance in filler sets. The result is that the estimated slopes for the two awareness groups might not have differed, although performance on repeated sets improved to different degrees compared with the baseline.

To test this possibility, we first conducted the analysis in the same way as it was done originally. The results of this analysis are presented in the top panels of Figure 4 and are labeled “Original: Free Intercept.” The reported Bayes factors reflect the evidence for the three-way interaction between trial type (repeated vs. filler), repetition, and awareness (aware vs. unaware), which is indicative of the difference in the Hebb repetition effect (i.e., the increase in immediate memory performance in repeated vs. filler lists over repetitions) between the two awareness groups.^6^ When analyzing the data in this way, the slope of the estimated learning effect does not credibly differ between the two awareness groups. Rather, all four experiments provide evidence against an effect of awareness, thereby replicating the original conclusions (although for two experiments the evidence remains inconclusive). In an alternative statistical model, we set the intercept for repeated and unrepeated memory sets to be equal to represent the fact that these conditions were indistinguishable in the first trial. This change in the analysis changed the results completely: All experiments now revealed overwhelming evidence in favor of an effect of repetition awareness on learning (see lower panels of Fig. 4 labeled “Updated: Fixed Intercept”).

The reanalysis demonstrates that the assumption of repetition learning not being affected by repetition awareness stands on shaky ground. When taking baseline performance into account, all three studies provide evidence that learning in the Hebb paradigm is strongly affected by participants’ awareness of the repetition.

### Is repetition awareness a necessary condition for repetition learning?

Showing that repetition learning correlates with participants’ repetition awareness still does not mean that awareness is required for learning to occur. To assert such a necessity, two criteria must be met: First, participants who report no repetition awareness should show no learning. Second, in those who report repetition awareness, awareness should precede any improvements in immediate recall performance and not just emerge as a consequence of the latter. If either condition is not met, repetition learning could still occur implicitly.

To test whether learning is observable for participants who report no repetition awareness, we again made use of the two large data sets introduced earlier (approximately 300 participants from a visual Hebb experiment and approximately 300 from a verbal Hebb experiment). In both studies, participants were asked at the end of the experiment whether they noticed the repetition of a particular memory set. This allowed us to split the data by participants’ repetition awareness and examine learning separately within the two awareness groups.

**Fig. 5.**
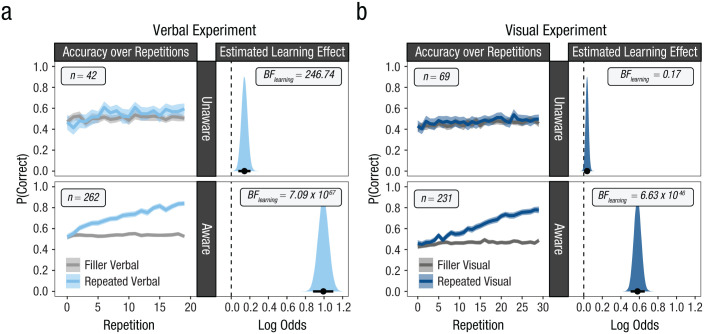
Large verbal and visual Hebb experiment. Data from this large (a) verbal and (b) visual Hebb experiment are split by a postexperimental assessment of participants’ repetition awareness. The left panels show the data plotted as a function of repetition aggregated over participants. Shaded areas reflect 95% confidence intervals. The right panels show the posterior distributions of the two-way interaction of trial type and repetition, which reflects an estimate of the learning effect. Points reflect the mean; bars represent the 95% highest density intervals. Parameters are estimated on the scale of log odds from a Bayesian hierarchical logistic regression model.

Figures 5a and 5b present the data from both experiments split by participants’ repetition awareness (left panels) alongside posterior estimates of the corresponding learning effects (i.e., the two-way interaction between trial type and repetition; right panels).^7^ Regarding the evidence for learning in the unaware group, the results provide a somewhat mixed picture. For the visual experiment, we found evidence against the presence of a learning effect in the unaware group, showing that no learning effect was observed without participants being aware of the repetition. This was different for the verbal experiment, in which the Bayes factor supported the presence of a learning effect, even in the absence of repetition awareness.

Does this imply that the Hebb repetition effect occurs implicitly? We consider this to be unlikely. Although our analysis cannot fully rule out that some learning might still occur implicitly, two points should be considered. First, the size of the estimated learning effect in the absence of awareness is very small, resulting in an almost negligible gain in immediate recall performance, even over many repetitions. Instead, a substantial Hebb repetition effect in the form of learning a whole repeated memory set as a new chunk (see previous section) seems to occur only in combination with explicit awareness. Second, the reliability of assessing repetition awareness on the basis of a postexperimental self-report can be questioned. For example, participants may misclassify themselves accidentally or because of different thresholds in reporting repetition awareness, which could potentially drive the observed small learning effect, even in the group of unaware participants.

Assessing awareness on the basis of a postexperiment questionnaire comes with another shortcoming: One cannot determine when in the experiment awareness arose. Concluding that awareness is a necessary condition for the Hebb repetition effect to occur, however, also requires showing that those participants who report awareness became aware of the repetition prior to any improvements in immediate recall performance, and not just in consequence. We recently addressed this issue by developing a new method that combines a Hebb paradigm with a trial-by-trial assessment of participants’ repetition awareness: After each trial, participants have to indicate whether they think that a just presented memory set had been presented before (repeated) or not (new). This allows assessing whether and at which point in the experiment participants are able to distinguish between a repeated memory set and unrepeated filler sets (for further details, see Musfeld et al., 2023).^8^ Combining this trial-by-trial assessment of participants’ repetition awareness with the trial-by-trial assessment of immediate memory performance within a joint modeling approach then allows us to estimate (a) the onset point of learning, (b) the onset point of repetition awareness, and (c) their temporal relation for each individual participant.

**Fig. 6.**
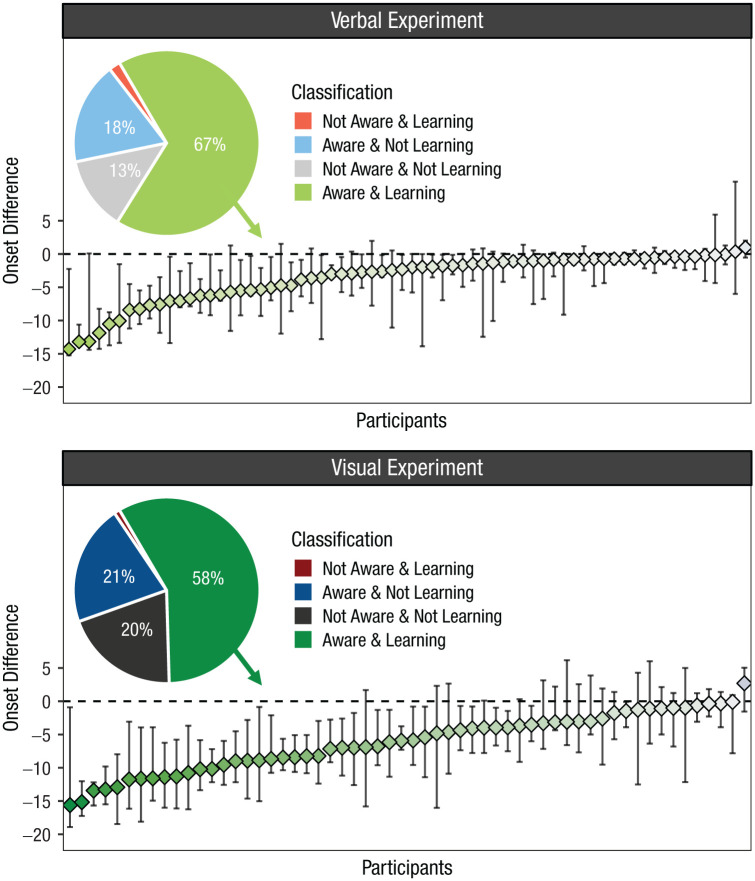
Results from jointly modelling a trial-by-trial assessment of participants’ repetition awareness and immediate recall performance. The pie charts show the cross-classification from classifying participants as aware and as learning, with the percentages for each category. Data points show the estimated difference between the onset of awareness and the onset of learning for each participant who was classified as aware and learning. Bars represent 95% highest density intervals. Negative differences indicate awareness prior to the onset of learning; positive differences indicate awareness after the onset of learning.

To illustrate this point, we once more applied this approach to the data from the visual Hebb experiment reported in Musfeld et al. (2023) as well as to the new data from the verbal Hebb experiment introduced here.^9^ Figure 6 shows the estimated differences between the onset of awareness and the onset of learning for each participant who was classified as learning and as becoming aware of the repeated memory set at some point. Negative differences indicate that the onset of awareness occurred prior to the onset of learning, whereas positive differences indicate the opposite. The results provide a clear picture: Across both experiments, and for every participant, repetition awareness either preceded or coincided with the onset of learning, showing that participants improved their recall performance only once they had recognized what was being repeated.

Taken together, the presented results show three important points: First, previous studies seem to have underestimated the effect of repetition awareness on repetition learning; second, learning effects are very small or absent altogether when no repetition awareness is reported; and third, measuring repetition awareness more objectively on a trial-by-trial level shows that recall performance improves only once participants explicitly recognize the repetition. This renders it unlikely that the Hebb repetition effect occurs implicitly. Rather, it suggests that explicit recognition of what is repeated is a necessary precondition for robust learning to occur (for earlier mentions of this idea, see also Bower & Winzenz, 1969; Cunningham et al., 1984; Winzenz, 1972). This demands an update of the proposed mechanisms underlying repetition learning. We sketch such an updated theoretical framework in the following section.

## Rethinking the Mechanisms Underlying Repetition Learning in the Hebb Paradigm—The Role of Episodic Memory

The benefit of repetitions on memory has not only been studied within the Hebb paradigm but also has motivated a large body of research within models of episodic memory (Benjamin & Tullis, 2010; Ensor et al., 2021; Hintzman, 2004, 2010; Hintzman & Block, 1971; Raaijmakers, 2003; Toppino & Gerbier, 2014). Episodic memory refers to our ability to store and recollect past experiences, including information about the temporal and spatial context an event has been experienced in (Renoult et al., 2019; Tulving, 1972, 1983). Most computational models of episodic memory are based on a form of instance theory (Jamieson et al., 2022; Logan, 1988, 2002), which postulates that every experienced event is stored as a separate record—or instance—within episodic memory, regardless of someone’s intention to remember (Craik & Tulving, 1975; Henke, 2010; Popov & Dames, 2023). Although this assumption closely aligns with what has been proposed in models of the Hebb repetition effect (Burgess & Hitch, 2006; Page & Norris, 2009), different proposals have been made regarding the representation of repeated experiences. Whereas some models have adopted the assumption that repeated experiences accumulate within a single trace in episodic memory (e.g., Gillund & Shiffrin, 1984; Murdock, 1995; Shiffrin & Steyvers, 1997; Wickelgren, 1972), an alternative approach has been formulated in the multiple-trace hypothesis (Hintzman & Block, 1971; Morton, 1968; Underwood, 1963). This hypothesis assumes that each experience is stored in an independent memory trace, even if it is a repetition. This facilitates memory for repeated events because it provides multiple independent access routes to the same information during retrieval (Hintzman, 1984; Lansdale & Baguley, 2008; Logan, 1988).

Although both approaches can account for a general benefit of repetition on memory, they have been criticized for various reasons (for a review, see R. L. Greene, 2008). On the one hand, empirical findings show that participants have access to information about separate occurrences of repeated items. This indicates that some independent memory trace must have been stored during each repetition, which contradicts the idea of a cumulative strengthening account (Hintzman, 2004, 2010; Hintzman & Block, 1971; Morton, 1968). On the other hand, empirical findings show that repetition effects on memory can be superadditive, which means that memory performance for repeated events is typically superior to what one would expect from an additive effect of independent memory traces (Begg & Green, 1988; Hintzman, 2004; Johnston & Uhl, 1976). This violates the assumption of independence in the multiple-trace hypothesis and suggests that repeated experiences have to strengthen their representations in memory in some interactive way (Benjamin & Tullis, 2010; Hintzman, 2010).

On the basis of the identified shortcomings, hybrid approaches have been proposed that combine ideas from multiple-trace and cumulative strengthening accounts. These approaches are based on the idea of study-phase retrieval (Thios & D’Agostino, 1976), which has also been referred to as “reminding” (Benjamin & Tullis, 2010; Hintzman, 2004, 2010; Tullis et al., 2014). Reminding accounts assume that a repetition is beneficial for memory only if a repeated encounter of an event is recognized as such and leads to the retrieval of a previous encounter during encoding (i.e., it “reminds” of having seen this before). If reminding is successful, previous and current experiences can be integrated, thereby cumulating both experiences within a strengthened memory trace. If a repetition does not “remind” of a previous encounter, however, the repetition will have no effect on memory traces of previous events, and rather an independent memory trace is formed (Benjamin & Tullis, 2010; Hintzman, 2004; see also Ensor et al., 2021; Shiffrin & Steyvers, 1997 for work on the third version of the retrieving effectively from memory model (REM.3), a version of the REM model that incorporates a similar mechanism). We now outline how such a reminding account offers a promising explanation for the Hebb repetition effect.

### A reminding account of the Hebb repetition effect

**Fig. 7.**
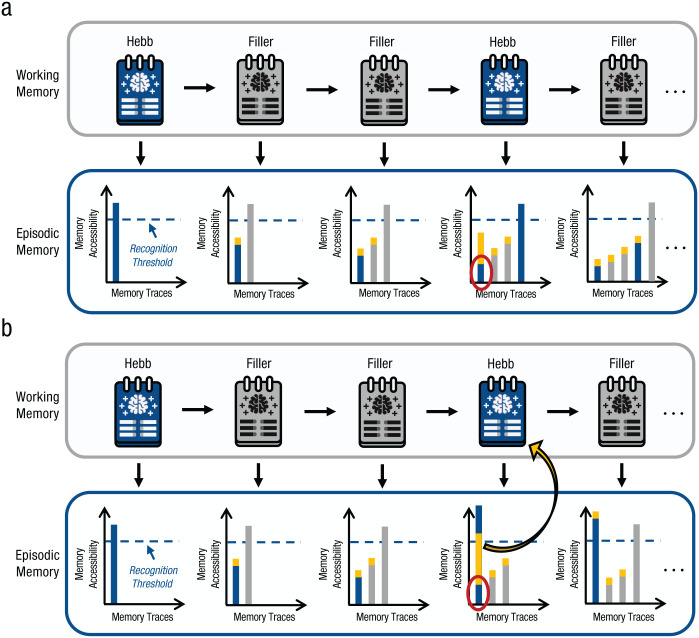
Schematic visualization of the reminding account of learning in the Hebb paradigm. Each encoded memory set leaves a trace in episodic memory with a given accessibility. Accessibility of previous traces is reduced by encoding new memory sets into memory. When a new memory is encoded, it can cue previous encounters in episodic memory, thereby increasing their accessibility (indicated by the orange bars on top of the memory traces). When a memory set is repeated, the repetition of that set could (a) fail to reactivate a previous encounter sufficiently to cause its explicit retrieval, in which case the repetition would not be recognized and an independent trace would be laid down in episodic memory; or (b) cue the successful retrieval of a previous encounter, thereby allowing one to integrate previous and recent encounters into a common trace and laying down a stronger trace instead of creating a new one.

The results reviewed above provide two crucial insights into the Hebb repetition effect: First, when data are analyzed on the level of individual participants, learning curves follow a two-stage process in which a phase without learning is followed by a rather rapid learning process, with an onset that varies between people; second, the onset of learning is always preceded by or coincides with the moment at which participants explicitly recognize that a memory set is presented repeatedly. This suggests that explicit recognition of what is being repeated is a necessary condition for learning to occur and is consistent with the previously described idea of “reminding.” When applying this concept to the Hebb repetition effect, one could sketch the process as follows (for a visualization, see Fig. 7).

We first adopt the assumption from instance theory that every event, like the presentation of a memory set in a memory experiment, creates a trace of this event in (episodic) memory.^10^ This idea has been consistently applied in both models of episodic memory and models of the Hebb repetition effect and builds the most basic prerequisite for repetition learning (Benjamin & Tullis, 2010; Burgess & Hitch, 2006; Gillund & Shiffrin, 1984; Hintzman, 1984; Logan, 2002; Shiffrin & Steyvers, 1997). Every time a new memory set is encoded, it will serve as a potential retrieval cue for previously encountered events stored in episodic memory. If the current event is a new memory set, it is unlikely to cue the retrieval of a previous event, and hence, a new independent trace is laid down in episodic memory. With that, multiple traces of similar memory episodes accumulate in episodic memory. When a memory set is repeated, two scenarios are possible.

First, encoding a repeated memory set could successfully cue the retrieval (or remind) of a previous encounter of the same memory set. In that case, a person explicitly recognizes the repetition and—rather than generating a new trace—the current experience is integrated into the previous trace that has been retrieved from episodic memory (Benjamin & Tullis, 2010). This creates a stronger version of the original memory trace that is likely to be even more accessible in future repetitions and thereby enables a rapid cumulative learning process. In essence, this scenario closely resembles what has been incorporated as a default assumption into the models of Burgess and Hitch (2006) and Page and Norris (2009) for every trial of a Hebb experiment.

However, a second scenario is possible that has not yet been considered for the Hebb repetition effect: Encoding a repeated memory set could fail to cue the retrieval of a previous encounter of the same memory set. In that case, the repetition is not recognized and will not result in learning. Instead, the episode is experienced as a new event and leaves an independent trace in episodic memory. If several repetitions of the same event remain unnoticed, multiple independent traces of this event accumulate in episodic memory. During this time, no performance improvements will be observed in the immediate memory task because knowledge does not accumulate within a single memory trace. However, storing multiple traces of the same event increases the probability that, at some point, at least one of these traces is retrieved during encoding of the repeated event and eventually results in learning (Hintzman, 1976; Hintzman & Block, 1971). This is consistent with the observation that when the onset of learning is delayed, it is still followed by a strong learning effect (Musfeld et al., 2023).

In the presented proposal, the success of repetition learning depends on the accessibility of memory traces for previous events in episodic memory: If an existing memory trace of a specific event is not accessible during its repeated encounter, memory representations are not strengthened. This leads to the prediction that the very same factors that have been identified to modulate the accessibility and strength of episodic memory traces should also modulate the success of repetition learning in the Hebb paradigm. Two of these factors that have been investigated extensively in research on episodic memory are the effects of retroactive interference and of retrieval practice, and we now discuss how the Hebb repetition effect is similarly affected by these variables.

### The role of retroactive interference for repetition learning in the Hebb paradigm

“Retroactive interference” refers to how newly memorized information can degrade the accessibility of previously memorized information, especially if that information has not yet been properly consolidated (Müller & Pilzecker, 1900; Wixted, 2021). In the Hebb paradigm, this effect can occur when the accessibility of a repeated episode is impaired by the encoding of several unique filler episodes in between two repetitions. In Figure 7, this is indicated by the decreasing accessibility of memory traces with the subsequent encoding of new memory episodes. This general idea was supported by an early finding by Melton (1963), who showed that learning effects in the Hebb paradigm can be impaired when more filler episodes are added in between repetitions.

The pure encounter of new events between repetitions, however, might not be sufficient to account for a possible delay or absence of repetition learning effects in the Hebb paradigm. As we established above, the occurrence of repetition learning effects depends on the successful recognition of a repeated memory set during its repeated presentation. In this process, the repeated presentation of a memory set serves as a (strong) retrieval cue that might still be sufficient to reactivate an episode of its previous encounter, even if its accessibility has been degraded. Thus, another important factor to consider is the specificity of the repeated memory set to act as a retrieval cue for reactivating a memory trace of its previous occurrence (Nairne, 2002). If all memory episodes are relatively distinct from each other, the repeated presentation of a memory set should offer a relatively specific cue, and thus the chance of recognizing a repetition should be high, even if several filler episodes have been encoded in between repetitions. If, however, memory episodes are highly similar, repeating a memory set provides a less specific cue and thus might fail to reactivate its previous encounter. This has also been referred to as “cue overload” and adds another form of interference to the retrieval stage of memories (Watkins & Watkins, 1975; Wixted, 2004). For the Hebb repetition effect, it leads to the prediction that learning should be harder with increasing similarity between lists.

Several studies have investigated the effect of list similarities on the Hebb repetition effect, typically by manipulating the degree of item overlap between repeated and filler lists (e.g., presenting the same items on every trial in a different order, or composing each list by different items; Dutli et al., 2024; A. J. Johnson & Miles, 2019; Melton, 1963; Page et al., 2013; Smalle et al., 2016). All of these studies align on the finding that higher list similarity/overlap resulted in a weaker Hebb repetition effect. This confirms the prediction described above and supports the assumption that repetitions have to be effective retrieval cues to reactivate memory traces of previous occurrences for repetition learning to occur. When this is not the case because many similar episodes have accumulated in episodic memory, repetitions can go unnoticed, leading to a delay or the absence of learning. This idea closely aligns with the mechanisms implemented in the model by Page and Norris (2009), which is also able to account for the effect of list similarities. It should be noted, however, that their model does not rely on episodic memory or the assumption that repetitions have to be recognized for learning to occur, thereby indicating that a contribution of episodic memory is not strictly implied by the list-similarity effect.

### The role of testing and retrieval practice for repetition learning in the Hebb paradigm

Research on episodic memory has shown testing to be effective in enhancing retention over the long term: Successfully retrieving a memory representation during a test increases its chances of being retrieved again on a future test (Carpenter et al., 2008; Roediger & Karpicke, 2006). Moreover, this effect is stronger if memory retrieval is more demanding—for instance in a recall test compared with a recognition test (Carpenter & Delosh, 2006; Karpicke & Roediger, 2007). Thus, testing is one way to create more accessible representations in episodic memory. In the Hebb paradigm, testing is inherent because each presented memory set is typically tested immediately after its presentation. Although this is primarily a test of working memory, it is still possible that representations in episodic memory contribute to this task such that individuals practice their retrieval and consolidate them in episodic memory (Bartsch & Oberauer, 2023; Oberauer & Awh, 2022; Oberauer et al., 2017; for evidence that episodic memory might contribute less to tasks typically used in the Hebb paradigm, however, see Oberauer & Bartsch, 2023). This leads to the prediction that testing, and especially the retrieval demands of the test, should influence the success of learning in the Hebb paradigm, most probably by affecting the accessibility of previously encoded episodes.

Several studies have investigated the effects of testing on the Hebb repetition effect, mostly focusing on the learning of verbal sequences (Cohen & Johansson, 1967; Cunningham et al., 1984; Kalm & Norris, 2016; Oberauer & Meyer, 2009). These studies compared conditions in which the presentation of a memory list was followed by an immediate serial recall test to conditions in which memory lists were only encoded but not tested. The results converged on the conclusion that testing is not necessary (Kalm & Norris, 2016; Oberauer & Meyer, 2009) but strongly facilitates the occurrence of (Cohen & Johansson, 1967; Cunningham et al., 1984; Kalm & Norris, 2016; Oberauer & Meyer, 2009) the Hebb repetition effect.

An even more critical role of testing has been identified for the Hebb effect in the visuospatial domain. Here, testing, and especially the need to recall the memory items, have been identified as key factors for the Hebb repetition effect to occur (Musfeld, Souza, & Oberauer, 2024; Souza & Oberauer, 2022). Whereas many previous studies have failed to observe a Hebb repetition effect in the visuospatial domain when working memory was tested by a recognition task (Fukuda & Vogel, 2019; Logie et al., 2009; Olson & Jiang, 2004), it is observed reliably when a recall test is used (Musfeld, Souza, & Oberauer, 2024; Souza & Oberauer, 2022). Furthermore, Souza and Oberauer (2022) showed that the strength of the Hebb repetition effect increases with the number of items that have to be recalled in each trial, thereby demonstrating a modulation of the effect by the retrieval demands at test.

Overall, these findings align with previous work on the testing effect (e.g., Carpenter & Delosh, 2006; Carpenter et al., 2008; Karpicke & Roediger, 2007; Roediger & Karpicke, 2006) in episodic memory. They imply that testing—and especially the retrieval demands of the test—provide an opportunity for individuals to practice the retrieval of the encoded information and to further consolidate that information in episodic memory, reducing its susceptibility to interference. This makes memory of the repeated sets more accessible and thereby more likely to be recognized and retrieved during a repeated encounter, hence increasing the probability of learning.

## Challenges and Future Directions

In the last section we sketched a tentative framework for explaining recent findings on the Hebb repetition effect that incorporates a critical involvement of episodic memory in repetition learning. Although we showed the plausibility of this account by highlighting many parallels between the Hebb repetition effect and research on episodic memory, there are also a few challenges that arise from this perspective.

One of these challenges is the observation of a Hebb repetition effect in amnesic patients with damages in the medial temporal lobe (e.g., Gagnon et al., 2004). The medial temporal lobe, and especially the hippocampus, have been strongly associated with functions of episodic memory (e.g., the recognition and retrieval of previously presented information), and damages in these brain areas have been found to result in related impairments (e.g., Eichenbaum et al., 2007; Kopelman et al., 2007; Manns et al., 2003; Squire et al., 2007; Wais et al., 2006). Thus, observing a Hebb repetition effect in patients with damages in the medial temporal lobe area brings into question the critical involvement of episodic memory in such learning. However, studies on this matter are rare, often rely on individual cases, and have so far led to mixed results. Whereas some studies found that repetition learning was preserved in amnesic patients (Baddeley & Warrington, 1970; Gagnon et al., 2004; Rausch & Ary, 1990), others observed related impairments (Charness et al., 1988; Drachman, 1966; Milberg et al., 1988). Thus, more direct tests of the involvement of episodic memory in the Hebb repetition effect will be required for testing the proposed account.

Another potential discrepancy lies in the relation between the Hebb repetition effect and the spacing effect in episodic memory. The spacing effect refers to the well-established finding that spaced repetitions (i.e., separating repeated study episodes by time and other content) leads to more successful learning than massed repetitions, without any separation of study opportunities (for overviews, see, e.g., Cepeda et al., 2006; Delaney et al., 2010; Toppino & Gerbier, 2014). The opposite, however, would be expected from the proposed framework: If the success of repetition learning depends on the accessibility of previous encounters of the same information in episodic memory, noninterleaved/massed repetition should result in more successful learning. Yet these relations are likely more complex than sketched within this simplified framework. The reminding model by Benjamin and Tullis (2010), for example, incorporates the assumption that reminding is more beneficial for learning when the associated information is more difficult to retrieve. This assumption could be easily integrated into the presented framework because it is consistent with the finding that the strength of the Hebb repetition effect increases with the retrieval demands during testing (e.g., Musfeld, Souza, & Oberauer, 2024; Souza & Oberauer, 2022). Thus, although massed repetitions in the Hebb paradigm might make the occurrence of successful remindings more likely, more spaced repetitions could still result in a stronger learning outcome over longer time intervals. This would be consistent with findings on the spacing effect, which typically show a dependency on the test delay (Cepeda et al., 2009; Pavlik & Anderson, 2005). One difficulty for this assessment is that the Hebb repetition effect is typically studied over only relatively short time intervals. Although it has been demonstrated that representations learned in the Hebb paradigm are stable over extended periods of time (Page et al., 2013), it is yet to be shown how the robustness of such learning might be influenced by different repetition spacings during the initial learning.

A final open question is whether repetition learning leads to a strengthening of representations in episodic memory or to the creation of new representations in semantic memory. In the presented framework, we adopted assumptions from reminding accounts, and more speciﬁcally, the assumption that repeated experiences are integrated into a previous episodic memory trace that is retrieved if reminding happens during encoding (Benjamin & Tullis, 2010). This allows this trace to become stronger over subsequent repetitions, but it keeps the locus of the learning effect within episodic memory. This also means that the retrieval of such representations remains dependent on episodic memory, which is an assumption that could be questioned. Learning from repetition is assumed to result in representations of knowledge, which is a kind of representation typically associated with semantic rather than episodic memory (Renoult et al., 2019; Tulving, 1972). Thus, an alternative could be that—once some repeated pattern is recognized—a new representation is formed, which only integrates, or abstracts, what is common among repeated study episodes. In this case, only the initial stage of the learning process (i.e., the recognition of what is repeated) depends on episodic memory, whereas the learned representation transitions into a state of semantic knowledge that is independent of speciﬁc contextual features an event has been experienced in. Future research will have to clarify to what extent a dependency on episodic memory remains throughout the learning process.

## Conclusion

The Hebb repetition effect has been one of the most influential phenomena for studying the cognitive processes underlying repetition learning and the exchange of information between working memory and long-term memory. In this article, we critically revisited the prevailing assumptions that have been proposed to explain learning in Hebb’s paradigm and reevaluated their validity in light of recent findings and developments. Contrary to long-standing assumptions, repetition learning in the Hebb paradigm neither occurs implicitly nor gradually with every repetition. These assumptions are likely an artifact from analyzing data aggregated over participants and do not resonate with the data patterns observed for individual participants. Instead, learning repeated patterns seems to require the explicit recognition of what is being repeated. Repetitions lead to a gradual buildup of stable representations in long-term memory only once a repeated pattern is identified. To account for these new insights, we outlined a theoretical framework based on models of episodic memory, and especially the idea of reminding. Future research will be needed to test the predictions of our account more critically and to develop a more formalized version of the account to derive more precise predictions. We believe that our proposal offers a new perspective that can explain many old findings and motivate new research on the topic.

## Supplemental Material

sj-docx-1-pps-10.1177_17456916251408052 – Supplemental material for Revisiting Hebb: The Mechanisms of Repetition Learning Supplemental material, sj-docx-1-pps-10.1177_17456916251408052 for Revisiting Hebb: The Mechanisms of Repetition Learning by Philipp Musfeld and Klaus Oberauer in Perspectives on Psychological Science

## Transparency

*Action Editor:* Arturo E. Hernandez

*Editor:* Arturo E. Hernandez

*Author Contributions*

**Philipp Musfeld:** Project administration; Conceptualization; Methodology; Software; Investigation; Formal analysis; Data curation; Validation; Visualization; Writing – original draft; Writing – review & editing.

**Klaus Oberauer:** Methodology; Resources; Funding; Supervision; Writing – review & editing.

**Open Science Practices:** All data and scripts related to the analyses presented in this article have been made publicly available on OSF and can be accessed at https://osf.io/p2xfe.

## Notes

1. We use the term “learning” to refer to an improvement in memory test performance, as shown, for instance, by the Hebb repetition effect.

2. Detailed R code for the simulation can be found at https://osf.io/p2xfe.

3. The data sets, Stan model, and a detailed analysis script for fitting that model to the data can be found at https://osf.io/p2xfe.

4. We did not use the data from the verbal Hebb experiment in Musfeld et al. (2023) because the testing procedure deviated from a typical Hebb task by using a cued random-order recall task instead of a serial recall task. To show that the same results generalize to the more typical scenario of the immediate serial recall task in the Hebb paradigm, we repeated the study using a serial recall procedure. Other than that, the design was the same as reported in Musfeld et al., 2023. The new data can be found at https://osf.io/p2xfe, and a detailed description of the method is provided in the Supplemental Material available online.

5. Because the original raw data were not available, we reconstructed the data from the information reported in the publications. We first extracted the mean proportion of correct responses within each design cell together with the total number of responses on which this average was based. This allowed us to calculate how many responses of the total number of responses were correct within each design cell and to feed that information into a binomial regression model. In this way, we were still able to take the number of participants and number of trials into account, although only averages were reported. Although this provides only an approximation of the data, we were able to replicate all conclusions reported in the original publication when using a similar analytical approach as reported therein. Detailed analysis scripts for reconstruction and analysis of the data can be found at https://osf.io/p2xfe. A more detailed description of the fitted models is provided in the Supplemental Material.

6. Further details about the analysis and the exact models that we fit are provided in the Supplemental Material.

7. For further details on the conducted analysis, see the Supplemental Material.

8. Note that this method inevitably points participants to the fact that there is a repeating memory set. This is information that is typically not provided to participants in a Hebb paradigm. To account for this, all reported experiments included control conditions to ensure that the additional knowledge did not affect the observed learning (see also the Supplemental Material).

9. This includes a subset of the two previously described data sets (approximately 100 participants each) for which we had collected trial-by-trial ratings of participants’ repetition awareness.

10. For simplicity, we assume that each memory set is stored within a single episodic memory trace, but the same idea could be applied to storing multiple traces for different subsets of a memory set. Under some conditions, encoding longer lists within smaller subsets might even increase the chance for detecting repeated patterns (see, e.g., Musfeld, Dutli, et al., 2024).
