# Supplementary material for: Revisiting Hebb: The Mechanisms of Repetition Learning
Source: Perspect Psychol Sci. 2026 Jan 30;21(3):229–50. doi: 10.1177/17456916251408052 (PMC13095083; doi:10.1177/17456916251408052)
Supplement: sj-docx-1-pps-10.1177_17456916251408052 – Supplemental material for Revisiting Hebb: The Mechanisms of Repetition Learning [file sj-docx-1-pps-10.1177_17456916251408052.docx]

**Supplementary Materials for:
“Revisiting Hebb: The Mechanisms of Repetition Learning”**

Philipp Musfeld^1,2^ & Klaus Oberauer^2^

^1^University of Amsterdam, Psychological Methods

^2^University of Zurich, Department of Cognitive Psychology

**Author Information**

Philipp Musfeld, Psychological Methods, University of Amsterdam, Netherlands, and Department of Cognitive Psychology, University of Zurich, Switzerland.

E-mail: [p.musfeld@uva.nl](mailto:p.musfeld@uva.nl)

Klaus Oberauer, Department of Cognitive Psychology, University of Zurich, Switzerland.

E-mail: [k.oberauer@psychologie.uzh.ch](mailto:k.oberauer@psychologie.uzh.ch)

Correspondence concerning this article should be addressed to Philipp Musfeld at [p.musfeld@gmail.com](mailto:p.musfeld@gmail.com).

**Open Science Practices**

All data and scripts related to the analyses presented here have been made publicly available on the OSF and can be accessed at <https://osf.io/p2xfe/>.

**Replication of Musfeld, Souza, and Oberauer (2023; PNAS) – Verbal Experiment**

For the purpose of this review, we conducted a full replication of the verbal Hebb Experiment reported in Musfeld et al. (2023). In the original study, we deviated from the typical Hebb paradigm by asking participants to recall the presented sequences of letters in a randomized order, cued by serial position. For the Hebb paradigm, however, it is more common to use a serial recall procedure. To ensure generalization of our results to a more typical Hebb paradigm, we repeated our experiment using the more common serial recall procedure. In the following, we report all necessary details on the study as well as some additional analyses.

Methods

*Participants*

Data was collected online via the participant platform Prolific. We recruited *N =* 309 participants, distributed over three experimental conditions: *n_no-information_* = 103, *n_information-only_* = 102, *n_awareness-rating_* = 104. All participants had to be between 18 and 35 years old, fluent in English, have no dyslexia, not participated in any of our lab’s previous Hebb experiments on Prolific, and an approval rate of at least 98% on Prolific. Additionally, we excluded participants who indicated at the end of the experiment to have 1) used aids to improve their memory performance throughout the experiment (e.g., by writing down the presented sequences), 2) not participated seriously in the experiment, or 3) who performed the experiment at chance level in Filler trials throughout at least one half of the experiment. Chance level was determined by using the 99^th^ quantile of the binomial distribution, given a guessing probability of 1/19 (the number of possible consonants), and a total number of 270 responses (9 responses per trial, 3 Filler trials per block, and 10 blocks per one half of the experiment). Overall, 5 participants were excluded due to these criteria, resulting in a final sample of *N* = 304 participants (*n_no-information_* = 103, *n_information-only_* = 100, *n_awareness-rating_* = 101).

*Stimuli*

Memory lists consisted of nine consonants which were sampled without replacement from the set of all consonants except “W” and “Y” because “W” is the only consonant containing more than one syllable and “Y” has the same function as a vowel in English language. Consonants were presented sequentially in the center of the screen with font size set to 8% of the vertical screen height.

All consonant lists were generated randomly anew upon starting the experiment but with the following constraints: 1) All stimulus lists needed to differ in at least three item-position combinations to avoid random duplication of Filler lists; 2) Filler lists were not allowed to share the first two consonants with the Hebb list; 3) Lists were not allowed to contain well known acronyms to avoid effects of semantic chunking. For this, a list of 81 well know 2- and 3-letter acronyms was collected (e.g., “PDF”, “BMW”, “CV”) and acronyms were not allowed to be part of any lists; 4) The consonants “M” and “N” were not allowed to be part of the same list because of their high degree of phonemic similarity.

*Design*

Upon starting the experiment, participants were randomly assigned to one of three between-subject conditions. The conditions differed in the instruction participants received at the beginning of the study: Participants in the *No Information* condition received no information about the possibility that memory sets can repeat; participants in the *Information Only* group were informed about this possibility; participants in the *Awareness Rating* condition were informed about the possibility of repetition and additionally asked to rate their awareness of a repetition after each trial. To assure instructions were read carefully, participants answered a short questionnaire about the experiment before proceeding to the main task. For the *Information Only* and the *Awareness Rating* group, this questionnaire contained a critical question about the possibility of repeating memory sets. Participants were only allowed to participate if all questions were answered correctly.

For each participant, a total of 80 memory lists was randomly created at the beginning of the experiment. One memory list was randomly selected as the repeated Hebb list, which was then repeated, on average, every 4^th^ trial, resulting in a total of 20 repetitions. For this, the experiment was divided into mini-blocks of four trials each. Within each mini-block, the Hebb list was shown once at a random position, with the only constraint that two Hebb lists were not allowed to follow immediately after another (see Figure S1A). The remaining trials involved the presentation of unique Filler lists.

Figure S1

Illustration of the experimental design. **A** General structure of the experiment, showing how the two trial-conditions are distributed across mini-blocks. **B** General flow of a single trial, illustrating the sequential presentation of consonants in the center of the screen.

*Procedure*

Figure S2B provides an overview of the flow of a trial. Each trial started with the presentation of a fixation cross in the center of the screen for 500 ms. Afterwards, the 9 consonants were presented sequentially in the center of the screen, with each consonant being visible for 500 ms, and a brief inter-stimulus-interval of 100 ms between consonants. Immediately after presentation of the last consonant, participants were prompted to recall the just presented sequence in its order of presentation. For this, a blinking prompt appeared in the center of the screen and participants typed their response by using their keyboard. Only consonants were accepted as valid inputs. When a consonant was input, the response was visible for 100 ms before prompting the next response. Omissions were not possible. When participants were not able to remember parts of the sequence, they were asked to make a guess. Once participants had recalled the whole sequence, they received a short information on how many consonants they had recalled correctly. Afterwards, participant in the *No Information* and the *Information Only* group moved on to the next trial. In the *Awareness Rating* condition, participants were asked if they had seen the just presented memory list before (*repeated*) or not (*new*). Participants responded by adjusting a visual slider scale, ranging from “*very certain new”* to *“very certain repeated”*. The center of the scale was labeled as *“uncertain”* and was used as the starting position of the slider.

After completing all trials of the working memory task, all participants were asked about their awareness of the repetition. In the *No Information* group, participants were first asked if they recognized anything specific about the experiment, and participants provided an open text response. Afterwards, they were told about the repetition of a specific memory list and asked if they had recognized it. In the other two conditions, participants were informed of list repetition prior to the experiment and only asked if they had noticed the repetition.

Overall, the experiment closely resembled the experiment by Musfeld et al. (2023), with the main difference that 1) all consonants were presented in the center of the screen (instead of a row of boxes that provided a visual outline of the list), and 2) memory was tested by a serial recall task (instead of recall in randomized order).

The Effect of Foreknowledge on Repetition Learning

Participants received different levels of foreknowledge about the possibility of memory list repetitions: Whereas participants in the *No Information* group were not informed about the possibility of memory list repetitions, participants in the *Information Only* and the *Awareness Rating* group were informed about this, and participants in the *Awareness Rating* group additionally rated their awareness of list repetitions on every trial. To test if this manipulation had any influence on learning, we tested if the observed learning effect differed between the three conditions. The data from the three conditions are presented in the left panels of Figure S2, showing immediate recall performance in Hebb and Filler lists as a function of repetition, aggregated over participants. We performed two analyses to assess potential differences between conditions.

Figure S2

Results from replicating the verbal Hebb experiment of Musfeld et al. (2023) with an immediate serial recall task. The three rows show the results split by the three experimental conditions. The plots on the left show memory performance as a function of repetitions, aggregated over participants. The plots in the middle and right panels show the results of the Bayesian hierarchical mixture model, fitted to the same data. The middle panels show the estimated learning curve for the three groups based on the shape of the underlying individual learning curves (i.e., combining the average onset point of learning with the average rate of learning). The right panels show the estimated mixture proportion, which reflects the proportion of participants who showed a learning effect in each group. Shaded areas around the aggregated and estimated learning curves reflect 95% within-subject confidence intervals and 95% highest density intervals respectively.

First, we fitted a Bayesian hierarchical logistic regression model to the data to assess condition differences in overall learning. This model included trial-type (*Hebb* vs. *Filler*), mini-block (i.e., the number of repetitions for the repeated Hebb list), and condition (*No Information* vs. *Information Only* vs. *Awareness Rating*) as predictors. Trial-type was dummy coded with *Hebb* = 1 and *Filler = 0*, so that Filler trials were used as a baseline. The number of repetitions was used as a continuous predictor, with the first mini-block set to 0, and the range of the values standardized to a standard deviation of 1. Condition was effect coded using orthonormal contrasts to ensure equal distribution of prior density to all possible pairwise condition differences (Makowski et al., 2019). This resulted in the following model equation:

$$\theta={logit}^{-1}(b_{0}+b_{1}*miniBlock+b_{2}*condition+b_{3}*miniBlock*condition$$

$$+ b_{4}*miniBlock*trialType+b_{5}*miniBlock*trialType*condition)$$

$$n_{correct}\sim Binomial(n_{responses}, \theta)$$

Here, the number of correct responses within a trial is modeled by a binomial distribution given the number of total responses within a trial and *θ,* which is the probability of a response being correct. *θ* is then modeled as a logistic function of the described predictor variables. In this setup of the model, the main effect of *miniBlock* reflects any changes in immediate memory performance in Filler trials, whereas the interaction between *miniBlock* and *trialType* reflects any changes in immediate memory performance in Hebb trials that is different from changes in Filler trials. Thus, this interaction term reflects the Hebb repetition effect. Consequently, the three-way interaction between *miniBlock, trialType,* and *condition* reflects any differences in this learning effect. The main effect of *trialType* as well as the two-way interaction between *trialType* and *condition* was omitted from the model to force the model to estimate the same intercept for Filler and Hebb trials. This equates the probability of giving a correct response in Filler and Hebb trials in the first mini-block because at its first presentation, the Hebb list is no different than a Filler list.

To test if there was any difference in learning between the three conditions, we computed a Bayes Factor for the three-way interaction between *miniBlock, trialType* and *condition* by comparing the full model to an alternative model omitting this interaction using *bridgesampling* (Gronau et al., 2020). Both models were estimated using R (R Core Team, 2023) and the R-package *brms* (Bürkner, 2020), and each model was run on 8 chains with 5000 post-warmup iterations each (40,000 iterations in total). Logistic priors with location = 0 and scale = 0.5 were used on all model parameters. This resulted in a Bayes Factor of *BF_01_* = 25.91 in favor of the model omitting the three-way interaction, thereby providing strong evidence against any difference in learning between the three experimental conditions.

To further investigate any possible differences in learning, we also applied the Bayesian hierarchical mixture model introduced in Musfeld et al. (2023) to the data and assessed the shape of the underlying learning function separately for each condition (see supplementary materials in Musfeld et al. (2023) for details on the model). The results are presented in the middle and left panels of Figure S2. The middle panels show the average shape of the estimated learning curve, and the right panels show the posterior distributions of the estimated mixture proportion (i.e., the proportion of participants who have been classified as learning). Again, the results show no difference between the three experimental conditions, ruling out an influence of the different levels of foreknowledge on the observed learning effect.

Further Analyses on the New Data Set

All further analyses on the new data set were conducted in the same way as reported in Musfeld et al. (2023), and the results are reported in the main article. Detailed scripts for these analyses can be found at <https://osf.io/p2xfe/>.

Reanalysis of the Effect of Awareness on Repetition Learning

In the main article we report a reanalysis of three previous studies that had investigated the relationship between repetition awareness and repetition learning in the Hebb paradigm (Couture & Tremblay, 2006; Guérard et al., 2011; McKelvie, 1987; see section “Assumption 3: Repetition Learning Occurs Implicitly”). Here we provide further details on the analyses.

As the original raw data were not available, we reconstructed the data from the information reported in the publications. We first extracted the mean proportion of correct responses within each design cell (i.e., within each combination of repetition, trial-type, and awareness group), together with the total number of responses this average was based on (i.e., the number of responses within each trial, multiplied by the number of trials and participants within each design cell). This allowed us to calculate how many responses out of the total number of responses were correct within each design cell, and to feed that information into a binomial regression model. In this way we were still able to take the number of participants and number of trials into account, although only averages were reported. While this provides only an approximation of the data, we were able to replicate all conclusions reported in the original publications when using a similar analytical approach as reported there.

For all analyses, we fitted Bayesian logistic regression models including three predictor variables and their interaction: trial-type (*Hebb* vs *Filler*), mini-block / repetition (i.e., the number of repetitions of the repeated Hebb set), and awareness group (*aware* vs. *unaware*). Trial-type was dummy coded with Hebb = 1 and Filler = 0, ensuring that Filler trials are treated as a baseline condition; repetition was entered as a continuous predictor starting at 0, and the range of values was scaled to a standard deviation of 1; condition was effect coded with *aware* = 1 and *unaware* = -1 to contrast any effects between these groups. The two presented analytical approaches only differed in which main effects and interaction terms were included into the model. For the first approach (termed “Original: Free Intercept”), we included all possible main effects and interactions, as has been usually done in previous studies. This resulted in the following model:

$$\theta={logit}^{-1}(b_{0}+b_{1}*miniBlock+b_{2}*trialType+b_{3}*awarenessGroup+$$

$$b_{4}* miniBlock*trialType+ b_{5}*miniBlock*awarenessGroup+$$

$$b_{6}*trialType*awarenessGroup+b_{7}*miniBlock*trialType*condition)$$

$$n_{correct}\sim Binomial(n_{responses}, \theta)$$

Here, the number of correct responses is modeled by a binomial distribution given the number of total responses and *θ*, which is the probability of a response being correct. *θ* is then modeled as a logistic function of the three predictor variables and their interactions. Given the coding of the predictor variables, the model parameters have the following interpretations:

- *miniBlock:* The average change in recall performance on Filler trials across mini-blocks / repetitions averaged over the two awareness conditions.
- *trialType:* The average difference in recall performance in Filler and Hebb trials in the very first mini-block (i.e., at the beginning of the experiment) averaged over the two awareness conditions.
- *awarenessGroup:* The difference in recall performance between the two awareness groups in Filler trials at the beginning of the experiment.
- *miniBlock x trialType:* The difference in the effect of mini-blocks / repetitions between Hebb and Filler trials, averaged over the two awareness groups.
- *miniBlock x awarenessGroup:* The difference in the effect of mini-blocks / repetitions on recall performance in Filler trials between the two awareness groups.
- *trialType x awarenessGroup:* The difference of the effect of trial-type in the very first mini-block between the two awareness groups
- *miniBlock x trialType x awareness Group:* The difference between the two awareness groups in how much the change in recall performance over mini-blocks / repetition differs between Hebb and Filler trials.

Two parameters are of particular interest: The two-way interaction between mini-blocks and trial-type as it reflects the Hebb repetition effect, namely the improvement in recall performance in Hebb trials over repetitions (i.e., the Hebb repetition effect) that goes over and above any improvement in the Filler trials; and the three-way interaction between mini-blocks, trial-type and the awareness group as it reflects the difference in the Hebb repetition effect between the two groups.

Whereas the full model is a valid approach of analyzing the data from a Hebb experiment, one fact has to be considered: Including the main effect of trial-type as well as the two-way interaction between trial-type and awareness group allows the model to estimate different intercepts (i.e., the recall performance at the beginning of the experiment) for Hebb and Filler trials in each awareness group. This can lead to an over- or underestimation of the Hebb effect (i.e., the two-way interaction between mini-block and trial-type) as we have illustrated in more detail in the main part of our manuscript. Thus, if these effects are included, learning is not solely reflected in the two-way interaction between mini-block and trial-type but can also be reflected in a main effect of trial-type. Consequently, when analyzing the effects of repetition awareness on repetition learning, one would also need to take the two-way interaction between trial-type and awareness group into account, which has been neglected in previous studies.

Alternatively, one can omit the main effect of trial-type and the interaction of trial-type and awareness group from the equation. This is what we did here, resulting in the following model (termed “Updated: Fixed Intercept”):

$$\theta={logit}^{-1}(b_{0}+b_{1}*miniBlock+b_{2}*awarenessGroup+$$

$$b_{3}* miniBlock*trialType+ b_{4}*miniBlock*awarenessGroup+$$

$$b_{5}*miniBlock*trialType*condition)$$

$$n_{correct}\sim Binomial(n_{responses}, \theta)$$

In this case, the model is forced to estimate the same intercept for Hebb and Filler trials, which equates performance in the two trial conditions at the beginning of the experiment. This is a reasonable assumption because at the beginning of the experiment, the Hebb list has only been presented once and therefore has the same status as a Filler list. It also ensures that any improvements in recall performance in the Hebb list over repetitions are estimated in reference to a common baseline (i.e., the performance in Filler lists), thereby purely reflecting the learning effect in the two-way interaction between mini-block and trial-type. Consequently, any differences in learning between the two awareness groups are now only reflected in the three-way interaction between mini-block, trial-type and the awareness group.

For the reported analyses, we fitted both the full and the restricted model to all data sets. Models were fit in a Bayesian framework using R (R Core Team, 2023) and the R-package *brms* (Bürkner, 2020), and ran on 4 cores with 15,000 post-warmup iterations each (60,000 in total). Logistic priors with location = 0 and scale = 0.7 were used on all model parameters. To quantify the evidence for an effect of repetition awareness on learning, we computed Bayes Factors in favor of the three-way interaction between mini-block, trial-type, and awareness group using the Savage-Dickey density ratio (Wagenmakers et al., 2010). The results for each data set and the two different models are reported in the main article.

Estimating the Learning Effect for Unaware Participants

In the main article we report an additional analysis on the two large data sets from a visual and a verbal Hebb experiment, in which we assess the evidence for a learning effect separately for the two awareness groups (and especially for the group of unaware participants). For this analysis we used the same model as described above (omitting the main effect of trial-type together with the interaction of trial-type and awareness group). The only difference was that we fitted the model as a hierarchical model, including mini-block and trial-type as random participant effects (this was not possible with the previous data sets). The model was run on 8 chains with 7,500 post-warmup iterations each (60,000 in total), and we used logistic priors with location = 0 and scale = 0.5 on all model parameters. From the full model, we then computed the conditional effects of learning in each of the two awareness groups (i.e., the two-way interaction between mini-block and trial-type within each awareness group). To test if the resulting posterior distributions credibly differed from 0, we computed Bayes Factors using the Savage Dickey density ratio. The results of this analysis are presented in the main article.

References

Bürkner, P.-C. (2020). Bayesian Item Response Modeling in R with brms and Stan. *arXiv:1905.09501 [Stat]*. http://arxiv.org/abs/1905.09501

Couture, M., & Tremblay, S. (2006). Exploring the characteristics of the visuospatial hebb repetition effect. *Memory & Cognition*, *34*(8), 1720–1729. https://doi.org/10.3758/BF03195933

Gronau, Q. F., Singmann, H., & Wagenmakers, E.-J. (2020). **bridgesampling**: An *R* Package for Estimating Normalizing Constants. *Journal of Statistical Software*, *92*(10). https://doi.org/10.18637/jss.v092.i10

Guérard, K., Saint-Aubin, J., Boucher, P., & Tremblay, S. (2011). The role of awareness in anticipation and recall performance in the Hebb repetition paradigm: Implications for sequence learning. *Memory & Cognition*, *39*(6), 1012–1022. https://doi.org/10.3758/s13421-011-0084-1

Makowski, D., Ben-Shachar, M., & Lüdecke, D. (2019). bayestestR: Describing Effects and their Uncertainty, Existence and Significance within the Bayesian Framework. *Journal of Open Source Software*, *4*(40), 1541. https://doi.org/10.21105/joss.01541

McKelvie, S. J. (1987). Learning and Awareness in the Hebb Digits Task. *The Journal of General Psychology*, *114*(1), 75–88. https://doi.org/10.1080/00221309.1987.9711057

Musfeld, P., Souza, A. S., & Oberauer, K. (2023). Repetition learning is neither a continuous nor an implicit process. *Proceedings of the National Academy of Sciences*, *120*(16), e2218042120. https://doi.org/10.1073/pnas.2218042120

R Core Team. (2023). *R: A language and environment for statistical computing* (Version 4.3.1) [Computer software]. R Foundation for Statistical Computing. https://www.R-project.org/

Wagenmakers, E.-J., Lodewyckx, T., Kuriyal, H., & Grasman, R. (2010). Bayesian hypothesis testing for psychologists: A tutorial on the Savage–Dickey method. *Cognitive Psychology*, *60*(3), 158–189. https://doi.org/10.1016/j.cogpsych.2009.12.001
